# Determinants of Mental Health Inequalities Among People With Selected Citizenships in Germany

**DOI:** 10.3389/ijph.2024.1607267

**Published:** 2024-08-27

**Authors:** Miriam Blume, Susanne Bartig, Lina Wollgast, Carmen Koschollek, Katja Kajikhina, Marleen Bug, Ulfert Hapke, Claudia Hövener

**Affiliations:** ^1^ Department of Epidemiology and Health Monitoring, Robert Koch Institute, Berlin, Germany; ^2^ Institute of Medical Sociology, Centre for Health and Society, Medical Faculty, Heinrich-Heine-University, Düsseldorf, Germany; ^3^ Institute of Sociology, Freie Universität Berlin, Berlin, Germany; ^4^ Department of Infectious Disease Epidemiology, Robert Koch Institute, Berlin, Germany

**Keywords:** migrant health, social determinansts of health, discrimination, depressive symptoms, anxiety

## Abstract

**Objectives:**

Mental health is essential for overall health and is influenced by different social determinants. The aim of this paper was to examine which determinants are associated with mental health inequalities among people with selected citizenships in Germany.

**Methods:**

Data were derived from the multilingual interview survey “German Health Update: Fokus (GEDA Fokus)” among adults with Croatian, Italian, Polish, Syrian, or Turkish citizenship (11/2021–05/2022). Poisson regressions were used to calculate prevalence ratios for symptoms of depression (PHQ-9) and anxiety disorder (GAD-7).

**Results:**

Sociodemographic (sex, income, age, household size) and psychosocial (social support and self-reported discrimination) determinants were associated with symptoms of depression and/or anxiety disorder. The prevalence of mental disorders varied most by self-reported discrimination.

**Conclusion:**

Our findings suggest mental health inequalities among people with selected citizenships living in Germany. To reduce these, social inequities and everyday discrimination need to be addressed in structural prevention measures as well as in interventions on the communal level. Protective factors (e.g., social support) are also important to reduce mental health inequalities on the individual and community level.

## Introduction

Depression and anxiety disorder are two of the most common mental disorders worldwide according to the World Health Organization [1]. Negative consequences of mental disorders include a lower quality of life and ability to participate in everyday life. Another consequence of depressive disorders can be earlier mortality due to an increased risk of suicide and comorbidities with other mental disorders, as well as an increased physical morbidity [2]. Symptoms of anxiety disorder include somatic complaints such as chronic pain, headaches, or insomnia [3]. Often, depression and anxiety disorder co-occur [2].

The development of mental disorders is based on multi-factorial processes in which genetic, psychological, and social factors interact. It is influenced by individual and structural risk (e.g., age, gender, poverty, traumatic events, discrimination) and protective factors (e.g., social support, being in a partnership), which strongly impact the development and progress of mental disorders [2, 4, 5]. A systematic review and meta-analysis showed that an individual’s socioeconomic position, especially income, is associated with depressive symptoms [6]. People with a personal or parental history of migration may experience additional risk factors that affect their health: Factors before, during, and after migration may play a relevant role such as reasons for migration, or host-country language proficiency [7].

It is important to consider the heterogeneity among people with a history of migration, in terms of working and living conditions, which have a major impact on health (e.g., access to healthcare, income, social support, and discrimination in labour and housing) [7]. Furthermore, it has been shown that discrimination in everyday life as well as structural and institutional discrimination are associated with worse mental health outcomes including depression and anxiety disorder [8]. Here it is important to consider that different reasons and dimensions of discrimination (e.g., due to racism, socioeconomic position, gender, age) mostly appear not as single factors but in interaction, as they are interdependent and intertwined. Therefore, multiple and intersectional discrimination is an important mental health determinant associated with specific social and health inequalities [9, 10].

In Germany, most analyses of mental health in migrant populations have focused on refugees (e.g. [11, 12]). However, large-scale studies examining the mental health of people with a history of migration, that enable differentiated analysis considering the heterogeneity of risk and protective factors in this population are lacking in Germany so far. The preliminary assumptions of this study were that specific factors, such as being female or having lower income, would be associated with higher levels of depression and anxiety symptoms, as indicated by prior research within the general population [2, 4]. Additionally, we anticipated that individuals with a history of migration would face specific challenges affecting their mental health, influenced by factors such as discrimination [13]. Thus, the research objectives in the present analyses were to investigate which sociodemographic, psychosocial, and migration-related determinants are associated with symptoms of depression and anxiety disorder among adults with selected citizenships (Croatian, Italian, Polish, Syrian, or Turkish) living in Germany, aiming to provide new insights by focusing on a broader migrant population and considering the heterogeneity of mental health factors within this population.

## Methods

### Study Design and Study Population

The study “German Health Update: Fokus (GEDA Fokus)” is a multimodal and multilingual interview survey among people with Croatian, Italian, Polish, Syrian, or Turkish citizenship aged 18–79 years living in Germany, conducted by the Robert Koch Institute (RKI). The study aimed to collect comprehensive information on the health status, health behaviour, living conditions, utilization of healthcare services, SARS-CoV-2 infections, and COVID-19 vaccination to enable differentiated analyses of associations with sociodemographic and migration-related factors [14].

Based on a sample of residents’ registration offices, individuals were randomly selected out of 99 cities and municipalities throughout Germany by the characteristic of citizenship (1st, 2nd, or 3rd citizenship; therefore, persons with dual citizenship were included). The selection of the five citizenships followed model calculations [14] using the foreigners’ statistics [15] and register movements [16] of the Federal Statistical Office from 2015 to 2017. The size of the citizenship groups, as well as the migration dynamics (inward and outward migration), were considered [14]. The study population included people between 18 and 79 years of age with Croatian, Italian, Polish, Syrian, or Turkish citizenship who had their main residence in one of the selected cities and municipalities at the time of data collection [14].

Data collection was carried out in a sequential mixed-mode design from November 2021 to May 2022. In a first invitation send by mail, study persons received login details for a web-based questionnaire either in German only or bilingual in German combined with one of the five study languages (Arabic, Croatian, Italian, Polish, and Turkish). Those study persons who neither responded nor declined to participate were offered to participate via a bilingual paper-based questionnaire containing the same questions send via mail with a first reminder letter. In a third contact step, a second reminder letter including the login details for the web-based questionnaire was sent out. Residents in larger cities who neither responded to the initial invitation nor to the first reminder letter were additionally announced home visits to conduct personal or telephone interviews with partly bilingual interviewers using the same questionnaire [14].

A total of 6,038 people (2,983 women and 3,055 men) participated in the survey GEDA Fokus. The response rate was 18.4% (Response Rate 1), according to the standards of the American Association for Public Opinion Research [17]. The study design of GEDA Fokus is described in more detail in the study protocol [14].

### Measurements

#### Dependent Variables

*Depressive symptoms* were assessed using the Patient Health Questionnaire-9 (PHQ-9) [18]. This standardized validated instrument consists of nine items addressing individuals’ subjective impairments during the last 2 weeks, with answer options ranging from “Not at all” (coded as 0) to “Nearly every day” (coded as 3). A sum score ranging from 0 to 27 was calculated, with higher scores indicating more depressive symptoms. Cases with one or more missing values were excluded. The variable was then dichotomized based on existing recommendations with a cut-off value of ≥10 indicating depressive symptoms (coded as 1) [19]. The validated instrument Generalized Anxiety Disorder 7 (GAD-7) including seven items, and comparable answer options were used to screen for *symptoms of anxiety disorder* within the last 2 weeks [20]. To categorize anxiety disorder, first, a sum score (range: 0–21) was computed, with higher scores indicating more anxiety symptoms. Cases with one or more missing values were excluded. Second, the score was dichotomized. Generalized anxiety disorder was defined by a score of ≥10 (coded as 1), as recommended [20].

#### Independent Variables

##### Sociodemographic Determinants

Males and females were classified according to the sex stated in their birth certificate (self-reported in the questionnaire). *Age* was categorized as either “18–39 years,” “40–59 years,” or “60–79 years.” To measure *level of education,* study participants educational and vocational qualifications were categorized into “low” (ISCED 0–2), “medium” (ISCED 3–4), and “high” (ISCED 5–8) according to the 2011 version of the International Standard Classification of Education (ISCED 2011) [21]. *Income* (net equivalized income) was calculated by considering the net monthly income of the household as a total, which could be indicated by an exact amount or a category, and the number and age(s) of household members. Missing income information was imputed using regression analytic procedures with information on age, sex, household size, education, employment status, occupational status, regional unemployment, and income tax information [22]. The income values were categorized as “low” (quintile 1), “medium” (quintile 2–4), and “high” (quintile 5). *Household size* was assessed by the question “What is the total number of people currently living in your household?”. People answering “I live alone” were coded as “single-person household.” Other responses indicating more than one person living in the household were categorized as “multi-person household.”

##### Psychosocial Determinants

*Social support* was quantified by the Oslo Social Support Scale (OSSS-3) [23], which consists of three items asking how many people a person can rely on (4-point scale), how much interest other people show in the person’s activities (5-point scale), and how easy it is to get help from neighbours (5-point scale). A score value was formed if all three questions were answered. The total sum score of the three questions was categorized into “low” [3–8], “medium” [9–11], and “strong” social support [12–14]. *Self-reported discrimination* was assessed using an adapted version of the Short Version of the Everyday Discrimination Scale (EDS), a five-item scale measuring exposure to life-time experiences of discrimination [24]. Respondents were asked whether they had experienced specific occurrences of interpersonal discrimination, such as receiving poorer service or being treated with less respect than other people in their daily life. Response options “very often,” “often,” “sometimes,” “rarely,” versus “never” were dichotomized. Self-reported discrimination was coded as “Yes” if at least one indication of “rarely” to “very often” occurred across the five scenarios. If all scenarios were answered with “never,” self-reported discrimination was coded as “No.” Cases with more than two missing values were excluded.

#### Migration-Related Determinants

*German language proficiency* included responses on native language (“German,” “another language”) and the self-assessed German language proficiency of those who did not state German as their native language. The response options were categorized into “native language/very good,” “good/moderate,” and “poor/very poor” [24]. *Duration of residence* was classified as “up to 10 years,” “11–30 years,” “31 years or more,” and “since birth” (for respondents born in Germany). *Experience of flight or persecution* was assessed by asking those who were not born in Germany the main reasons for migration to Germany. The reasons “I have moved to Germany because there is/was war in my country” or “I moved to Germany because I was persecuted in my country (e.g., for political or religious reasons or due to my sexuality)” were summarized as “Yes”; other answers and participants born in Germany were categorized as “No.”

### Statistical Analysis

First, the prevalence of symptoms of depression and anxiety disorder were determined. Second, Pearson’s chi-squared tests of independence with the Rao-Scott second-order correction were performed to test for differences between groups. Third, prevalence ratios (PRs) with 95% confidence intervals (CIs) were calculated using Poisson regression models for each outcome (symptoms of depression and anxiety disorder), along with the sociodemographic, psychosocial, and migration-related determinants. Variables with significant associations in bivariate analyses were integrated into the multivariable Poisson regression models. German language proficiency was also added to the models where relevant per the literature. All regression models were adjusted for mode of survey administration and the citizenship according to residents’ registration offices. Results were considered significant with a level of uncertainty of <0.05.

We conducted complete case analyses and hence, excluded all participants with at least one missing value in the presented sociodemographic, psychosocial and migration-related as well as in the outcome variables, resulting in 5,640 cases analysed for symptoms of depression and 5,651 cases analysed for symptoms of anxiety disorder. A weighting factor was applied in the analyses to align the sample with the population of corresponding citizenships using the following characteristics: region, sex, age, education (ISCED 2011) and duration of residence [14]. These marginal distributions were taken from the 2018 Microcensus [25] after narrowing the data to the selected five citizenship groups (including dual citizenship). The analyses were performed using Stata 17.0 (Stata Corp., College Station, TX, United States).

## Results

The study population of GEDA Fokus included a total of 6,038 participants. As shown in Table 1, the prevalence for depressive symptoms according to the PHQ-9 was 21.3% based on the answers of 5,640 people. Of the 5,651 study participants analysed for the GAD-7, 13.4% reported symptoms of an anxiety disorder. The prevalence of both symptoms in the bivariate analyses varied by sex, age, household size, social support, self-reported discrimination, and duration of residence: Females, younger participants, those living in single-person households, as well as those experiencing lower social support and discrimination reported both symptoms more often. People living in Germany since birth showed a higher prevalence of depressive symptoms compared to those participants living in Germany for 31 years and longer. The prevalence of symptoms of anxiety disorder were higher for people with a duration of residence up to 10 years. Depressive symptoms were reported more often by people with experience of flight or persecution and low income compared those with high income (see Table 1).

**TABLE 1:** Symptoms of depression (n = 5,640) and anxiety disorder (n = 5,651) by sociodemographic, psychosocial, and migration-related determinants. German Health Update: Fokus (GEDA Fokus), (Germany. 2021–2022).

	Depressive symptoms (PHQ-9)			Symptoms of anxiety disorder (GAD-7)		
	n^a^	% (95% CI)^b^	*p*-value	n^a^	% (95% CI)^b^	*p*-value
Total	1,210/5,640	21.3 (19.41–23.34)	—	859/5,651	13.4 (11.94–15.09)	—
Sociodemographic determinants						
Sex						
Male Female	578/2,865 632/2,775	18.6 (16.21–21.35) 24.5 (21.79–27.40)	**0.003**	389/2,869 470/2,782	11.1 (9.27–13.33) 16.2 (14.17–18.41)	**<0.001**
Age groups						
18–39 years	745/2,913	25.5 (22.58–28.54)	**<0.001**	534/2,920 255/1,996 70/735	16.4 (14.04–18.98) 11.3 (9.11–13.83) 9.3 (6.71–12.74)	**<0.001**
40–59 years	366/1,994	18.6 (15.71–21.83)				
60–79 years	99/733	14.7 (11.48–18.65)				
Education (ISCED)						
Low Medium High	361/1,528 476/2,147 373/1,965	23.2 (19.86–26.85) 20.3 (17.90–22.87) 18.7 (15.30–22.70)	0.152	244/1,541 327/2,143 288/1,967	13.7 (11.10–16.67) 13.6 (11.48–15.98) 12.5 (9.94–15.49)	0.832
Income						
Low Medium High	272/1,009 719/3,244 219/1,387	28.4 (23.64–33.68) 21.7 (19.39–24.13) 13.4 (10.59–16.80)	**<0.001**	178/1,013 503/3,253 178/1,385	16.2 (12.37–20.92) 13.2 (11.17–15.62) 11.4 (8.67–14.71)	0.188
Household size						
Single-person household Multi-person household	289/1,016 921/4,624	29.7 (24.82–35.13) 19.8 (17.82–22.00)	**<0.001**	212/1,021 647/4,630	21.9 (17.47–27.10) 12.0 (10.35–13.75)	**<0.001**
Psychosocial determinants						
Social support (OSSS-3)						
Low Medium High	516/1,422 537/2,913 157/1,305	36.5 (32.37–40.75) 18.8 (16.14–21.67) 11.3 (8.51–14.82)	**<0.001**	357/1,426 387/2,918 115/1,307	20.7 (17.63–24.12) 12.8 (10.73–15.13) 7.6 (5.63–10.11)	**<0.001**
Self-reported discrimination						
Yes No	1,099/4,133 111/1,507	26.8 (24.48–29.24) 7.5 (5.44–10.23)	**<0.001**	779/4,142 80/1,509	16.5 (14.66–18.57) 5.7 (4.15–7.85)	**<0.001**
Migration-related determinants						
Duration of residence						
Since birth Up to 10 years 11–30 years 31 years and longer	292/1,158 552/2,351 177/957 189/1,174	24.4 (20.57–28.62) 23.7 (19.98–27.76) 22.0 (17.85–26.72) 16.3 (13.27–19.78)	**0.013**	178/1,161 424/2,357 128/956 129/1,177	13.7 (10.92–17.00) 16.4 (13.06–20.48) 13.9 (10.90–17.51) 9.9 (7.43–13.15)	**0.035**
German language proficiency						
Native language/very good Good/moderate Poor/very poor	506/2,477 584/2,657 120/506	21.0 (18.50–23.69) 21.7 (18.86–24.79) 21.1 (15.86–27.44)	0.911	361/2,486 412/2,657 86/508	14.0 (11.89–16.32) 12.8 (10.61–15.37) 14.3 (9.73–20.39)	0.712
Experience of flight or persecution						
Yes No	322/1,147 888/4,493	28.5 (23.37–34.21) 19.8 (17.68–22.14)	**0.004**	225/1,157 634/4,494	17.4 (12.65–23.50) 12.6 (11.00–14.39)	0.078

Note. 95% CI, 95% confidence interval; ISCED, international standard classification of education; OSSS-3, Oslo social support scale; Significant associations based on the chi-squared test in bold.

^a^ n = unweighted number of participants.

^b^ % = weighted percentage.

**FIGURE 1:**
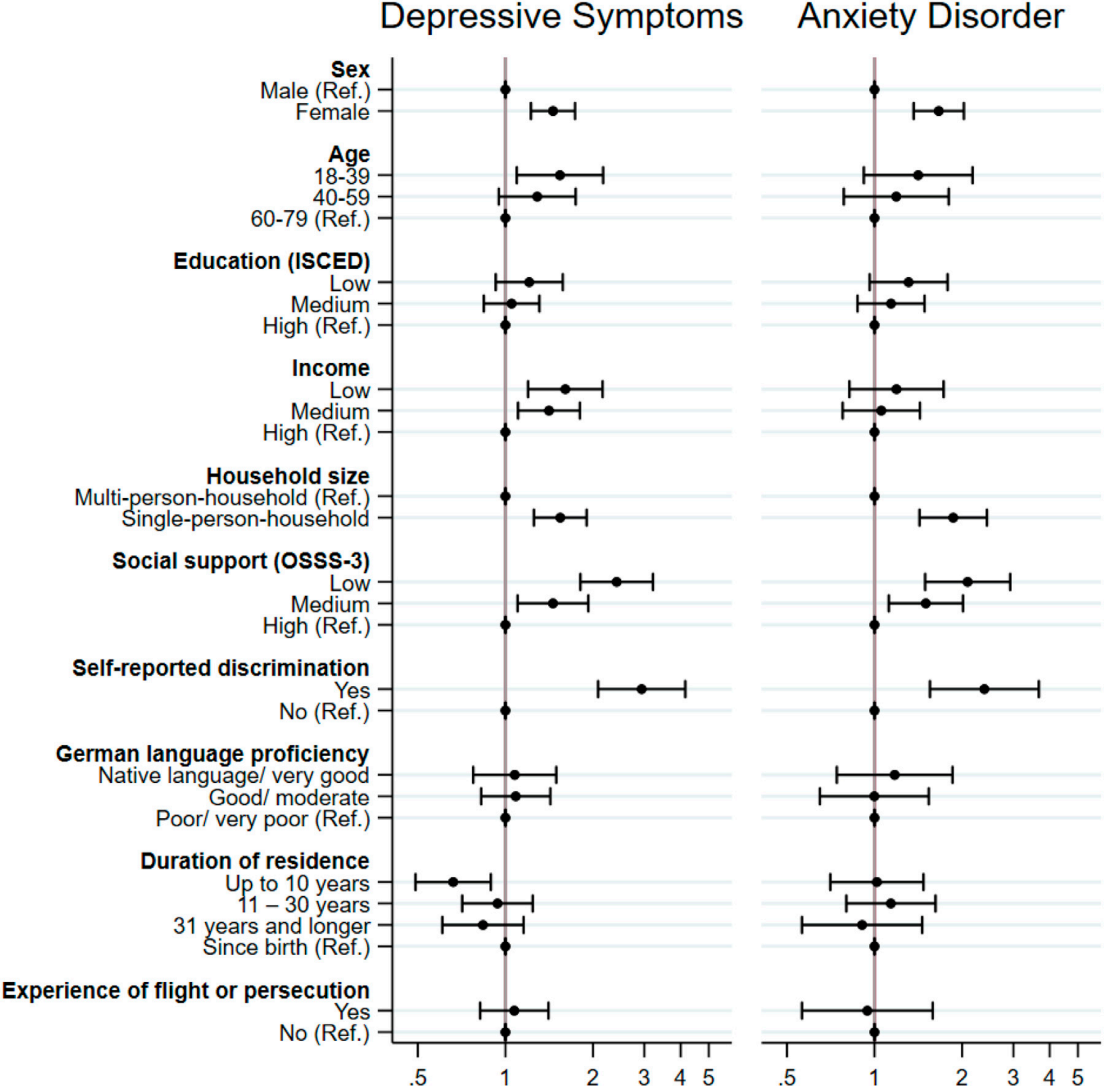
Prevalence ratios and 95% confidence intervals for symptoms of depression (n = 5,640) (PHQ-9) and anxiety disorders (n = 5,651) (GAD-7) by sociodemographic, psychosocial, and migration-related factors—results of Poisson regression analysis. German Health Update: Fokus (GEDA Fokus), (Germany. 2021–2022). Note. Adjusted for mode of survey administration and citizenship according to residents’ registration offices.

The results from the multivariable regression models show that the prevalence of symptoms of depression and anxiety disorder were higher for females (PR depressive symptoms = 1.46 (95% CI: 1.22–1.73); PR symptoms of anxiety = 1.66 (95% CI: 1.36–2.03), those living in single-person households [PR depressive symptoms = 1.54 (95% CI: 1.25–1.90); PR symptoms of anxiety = 1.86 (95% CI: 1.43–2.43)], those reporting low or medium social support [PR depressive symptoms = 2.41 (95% CI: 1.81–3.21), 1.46 (95% CI: 1.10–1.93); PR symptoms of anxiety = 2.09 (95% CI: 1.49–2.92), 1.50 (95% CI: 1.12–2.01)], and for those reporting experiences of discrimination [PR depressive symptoms = 2.94 (95% CI: 2.08–4.15); PR symptoms of anxiety = 2.38 (95% CI: 1.55–3.67). People with a duration of residence of up to 10 years reported symptoms of depression less often compared to participants born in Germany [PR depressive symptoms = 0.66 (95% CI: 0.49–0.89)] (see Figure 1; Supplementary Table A1). Those of a younger age [PR = 1.54 (95% CI: 1.09–2.17)] as well as those with a low or medium income [PR = 1.61 (95% CI: 1.20–2.16), 1.41 (95% CI: 1.11–1.80)] reported a higher prevalence of depressive symptoms but not symptoms of anxiety disorder.

## Discussion

This study aimed to identify which sociodemographic, psychosocial, and migration-related determinants are associated with an increased prevalence of symptoms of depression and anxiety disorder. Our findings suggest that there are inequalities in the distribution of mental health among people with selected citizenships, in particular due to sociodemographic and psychosocial determinants, with discrimination having shown the highest variety. In a sample of the general population in Germany in 2020 using the PHQ-8 the prevalence of depressive symptoms was 7.6% and thus lower than in our sample [26]. Also, for symptoms of anxiety disorder, a nation-wide study focusing on adults living in Germany showed a lower prevalence in different years using the two-item GAD-2 questionnaire (6.7%–9.6% between 2017 and 2021) [27]. Potential explanations for the higher prevalence of depressive and anxiety symptoms in our study are the timing - our study was conducted at a later stage of the COVID-19 pandemic - the differences in measurement, but also the sample composition. In Germany the risk of poverty is higher in people with a non-German compared to German citizenship [28], and poverty is a risk factor for adverse mental health outcomes [7, 8]. Other explanatory factors include the high prevalence of everyday discrimination and factors associated with the migration process [7, 8].

### Sociodemographic Determinants of Mental Disorders

The sex differences in mental disorders in our analyses are in line with previous research on the general population, indicating that women reported symptoms of mental disorders more often than men [29]. Various reasons for these differences between women and men are discussed: biological (e.g., hormonal reactions, genetic factors), psychological (e.g., body shame and dissatisfaction, rumination, and co-rumination), and micro-level (e.g., gender-based violence, intimate partner violence, work-family conflicts) and macro-level factors (e.g., gender discrimination, societal structural gender inequities) [30]. Regarding the effect of income, our results are consistent with a meta-analysis that showed a higher risk of depressive symptoms for adults with lower income [6]. Having a lower income can lead to strain due to fewer opportunities for participation, constant worry about resources, and a lack of the basic supplies for life [4]. Furthermore, our finding that younger people reported depressive symptoms more frequently has also appeared in the literature [2, 31]. The age differences in depressive symptoms can be partly explained by different situations in life people live in, like economic or social differences. Younger people are, for example, more likely to experience economic hardship and negative interpersonal exchanges, which in turn explain the more frequently reported depressive symptoms. Older people, conversely, are usually more economically secure and consciously avoid negative interpersonal exchanges [31]. Furthermore, our analyses revealed that individuals living alone had a higher risk of depressive or anxiety symptoms. Household size is associated with social inclusion or isolation, as loneliness is more common among people living alone [32]. It is particularly important to investigate the role of living alone in mental disorders, as the number of single-person households has increased over time in Europe, including Germany [33].

### Psychosocial Determinants of Mental Disorders

The highest prevalence of depressive and anxiety symptoms was reported by people with experiences of discrimination compared to other determinants. This is in line with other studies summarized in a review analysing research between 1983 and 2013, which showed that experiences of discrimination negatively affect mental health as well as physical health [13]. Nancy Krieger who developed the *ecosocial theory* highlights that discrimination leads to the embodiment of social inequalities and to chronic biological and physiological processes that result in reduced immune function, higher susceptibility for infections and ultimately in poorer overall health leading to increasing health disparities [34]. Additionally, research shows that discrimination on the interpersonal, institutional or structural level can hinder social mobility, social participation, which are all factors associated with poorer mental health outcomes [34]. The relationship between social support and mental health is well known [35]. Our results are consistent with a previous review among the general population, showing that a high level of social support is associated with a lower risk for symptoms of depression and anxiety disorder [2, 36]. A lack of social support and feelings of loneliness are linked to mental stress [37].

### Migration-Related Determinants of Mental Disorders

In contrast to sociodemographic and psychosocial determinants, most of the included migration-related determinants showed no association with symptoms of depression or anxiety disorder in our multivariable analyses. Our findings indicated a lower prevalence of depressive symptoms with a duration of residence shorter than 10 years. On the one hand, past research has discussed that post-arrival challenges, such as language barriers, uncertain residence status, shared accommodation, lack of a work permit or challenges in navigating within a new social environment, and barriers to the healthcare system, can have a direct impact on health [38]. However, this tends to contradict our finding that a shorter duration was a protective factor. On the other hand, after a longer duration of residence, the people who initially arrived with hopes and aspirations in a new country can experience disillusionment and be exposed to permanently increased psychological stress due to persistent participation obstacles [38]. For example, the poorer average housing and working and living conditions of people in Germany after migration show that these disadvantages make a significant contribution to health [8]. Moreover, experiences of discrimination, racism, and disadvantages on an institutional and structural level are discussed; these conditions and their effects on health change over the course of the (migration) biography [38]. These permanent burdens and disadvantages can reduce possible positive aspects of health over time and may explain the change of a duration of residence of longer than 10 years being no longer a protective factor. Also, to compare our results with the contradicting results in the literature, our unique sample needs to be considered, which is further discussed in the limitations section. Further multivariable analyses, for example, examining the associations between structural and institutional discrimination and mental health outcomes, are therefore necessary to examine the observed effects and differences in the present sample in relation to the co-factors described here.

### Strengths and Limitations

The interview survey GEDA Fokus and therefore our analysis, represent a significant addition to the national research landscape, as it examines the mental health of a large group of people with a history of migration on the basis of a variety of socio-demographic, psychosocial and migration-related determinants. This paper provides new insights by focusing on a broader migrant population and by considering the heterogeneity of mental health factors within this population. However, there are some limitations to consider when interpreting the results. We note that the differentiation between the concepts of depressive symptoms (PHQ-9) and symptoms of anxiety disorder (GAD-7) were seen to partially overlap in a validation study, and therefore no 100% separation of both concepts can be assumed. Nevertheless, it has been indicated that the differentiation of both concepts is sufficient [20]. Furthermore, GEDA Fokus was a cross-sectional study, therefore no causal explanations on the direction of the interrelationship between mental health outcomes and the determinants investigated are possible. Hence, it might be conceivable as well, that people with mental health disorders more often report lower levels of social support or experiences of discrimination instead of reporting more mental disorders because of having low social support or experiencing discrimination. Longitudinal studies are needed to get more in-depth insights into the causal relations between the observed associations. Additionally, the sample was recruited on the basis of citizenship, hence, individual subgroups, such as naturalized people with exclusively German citizenship, were not included, nor were people with other citizenships. Using register-based sampling might additionally systematically exclude certain groups within the population, such as people without a legal immigration status or those not registered at the resident’s registration offices. Hence, generalizability of our results is restricted. Also, due to our mixed-mode design, interviewer bias was possible, in particular with sensitive topics like mental health and experiences of discrimination. It is known, that questions on psychological aspects are answered differently, and probably more honest, in self-administered survey modes compared to interviews due to social desirability bias [39]. However, we addressed this issue by adjusting for mode of survey administration in the regression models. However, the sequential multilingual mixed-mode design supported the inclusion of different, especially hard to survey subgroups, such as older people, and people with lower levels of income, education, or subjective health [40].

### Conclusion

Our findings suggest mental health inequalities among adults with Croatian, Italian, Polish, Syrian, or Turkish citizenship living in Germany. The greatest variety of symptoms of depression and anxiety disorder was found related to experiences of discrimination highlighting that discrimination contributes to health inequalities. To reduce these mental health inequalities, social inequities and everyday discrimination need to be addressed because they can hinder participation, for instance, in healthcare or the labour market, which can have a great impact on symptoms of depression and anxiety disorder [8]. With this aim, experiences of discrimination, racism, and disadvantages on an institutional and structural level need to be addressed on the policy level [8]. Targeted anti-discrimination programs should be implemented nationwide at the workplace, in public health services, and at research institutions, that improve protection against discrimination and focus on the right to equal treatment as well raising diversity-sensitivity. Additionally, enabling better access to counselling and support services can be helpful for people experiencing discrimination but also for the documentation and collection of anti-discrimination data [41]. To make services accessible, it is necessary that they are multilingual, available close to home, and that they are sufficiently known about [42]. In addition, promoting protective factors, such as social support, can help to reduce the risk of mental disorders [2, 43]. For this, it is important to encourage social exchange and interaction with friends, relatives, and peers, and to support interventions, such as social work, that strengthen social support [44]. One group that should be considered in particular are people living in single-person households, who reported more symptoms of depression and anxiety disorder in our sample. Potential interventions to promote mental health in this population are educational and social activity group interventions in close proximity to peoples’ homes [45]. To observe how social inequalities in (mental) health develop overtime and which factors play a role therein, long-term monitoring is necessary. This enables both the observation of trends and effects of acute crisis situations (such as the pandemic) on mental health and the sustainable assessment of mental health determinants to derive effective and targeted public health measures.
