# Supplementary material for: Determinants of Mental Health Inequalities Among People With Selected Citizenships in Germany
Source: Int J Public Health. 2024 Aug 27;69:1607267. doi: 10.3389/ijph.2024.1607267 (PMC11383781; doi:10.3389/ijph.2024.1607267)
Supplement: Supplementary file 1 [file Table1.pdf]

SUPPLEMENTARY MATERIAL

Determinants of Mental Health Inequalities Among People With Selected Citizenships in Germany

Supplementary Table A1. Prevalence ratios and 95% confidence intervals for symptoms of depression (n = 5,640) (PHQ-9) and anxiety disorders (n = 5,651) (GAD-7) by sociodemographic, psychosocial, and migration-related factors – results of Poisson regression analysis. German Health Update: Fokus (GEDA Fokus), (Germany. 2021–2022).

	**Depressive symptoms (n = 1,253/ 5,640)**			**Symptoms of anxiety disorder (n = 893/ 5,651)**		
	**PR**	**95% CI**	**p-value**	**PR**	**95% CI**	**p-value**
**Sociodemographic determinants**						
**Sex**						
Male	Ref.			Ref.		
Female	1.46	1.22–1.73	**<0.001**	1.66	1.36–2.03	**<0.001**
**Age groups**						
18–39 years	1.54	1.09–2.17	**0.014**	1.41	0.92–2.17	0.114
40–59 years	1.29	0.95–1.74	0.104	1.19	0.78–1.80	0.412
60–79 years	Ref.			Ref.		
**Education (ISCED)**						
Low	1.21	0.93–1.57	0.162	1.31	0.96–1.78	0.085
Medium	1.05	0.84–1.31	0.659	1.14	0.87–1.49	0.329
High	Ref.			Ref.		
**Income**						
Low	1.61	0.20–2.16	**0.002**	1.19	0.82–1.73	0.356
Medium	1.41	1.11–1.80	**0.006**	1.06	0.78–1.43	0.728
High	Ref.			Ref.		
**Household size**						
Single-person household	1.54	1.25–1.90	**<0.001**	1.86	1.43–2.43	**<0.001**
Multi-person household	Ref.			Ref.		
**Psychosocial determinants**						
**Social support OSS-3)**						
Low	2.41	1.81–3.21	**<0.001**	2.09	1.49–2.92	**<0.001**
Medium	1.46	1.10–1.93	**0.009**	1.50	1.12–2.01	**0.007**
High	Ref.			Ref.		
**Self-reported discrimination**						
Yes	2.94	2.08–4.15	**<0.001**	2.38	1.55–3.67	**<0.001**
No	Ref.			Ref.		
**Migration-related determinants**						
**Duration of residence**						
Since birth	Ref.			Ref.		
Up to 10 years	0.66	0.49–0.89	**0.007**	1.02	0.71–1.47	0.919
11–30 years	0.94	0.71–1.24	0.566	1.14	0.80–1.62	0.467
31 years and longer	0.84	0.61–1.15	0.208	0.91	0.56–1.46	0.682
**German language proficiency**						
Native language/very good	1.08	0.78–1.50	0.675	1.17	0.74–1.85	0.490
Good/average	1.09	0.83–1.43	0.590	1.00	0.65–1.54	0.996
Poor/very poor	Ref.			Ref.		
**Experience of flight or persecution**						
No	Ref.			Ref.		
Yes	1.07	0.82–1.41	0.606	0.94	0.56–1.58	0.828

Note. PR = prevalence ratio; 95% CI = 95% confidence interval; ISCED = International Standard Classification of Education; OSS-3 = Oslo Social Support Scale.
